# A Bietti Crystalline Dystrophy Mouse Model Shows Increased Sensitivity to Light-Induced Injury

**DOI:** 10.3390/ijms232113108

**Published:** 2022-10-28

**Authors:** Zhiwei Ma, Xiaodong Jiao, Martin-Paul Agbaga, Robert E. Anderson, Haohua Qian, Qian Li, Lijin Dong, J. Fielding Hejtmancik

**Affiliations:** 1 Ophthalmic Genetics and Visual Function Branch, National Eye Institute, National Institutes of Health, Bethesda, MD 20892, USA; 2 Departments of Ophthalmology, Cell Biology, Oklahoma Center for Neuroscience, Harold Hamm Diabetes, and Dean McGee Eye Institute, University of Oklahoma Health Sciences Center, Oklahoma City, OK 73104, USA; 3 Visual Function Core, National Eye Institute, National Institutes of Health, Bethesda, MD 20892, USA; 4 Beijing Ophthalmology & Visual Sciences Key Lab, Beijing Tongren Eye Center, Beijing Tongren Hospital, Capital Medical University, Beijing 100051, China; 5 Genetic Engineering Core, National Eye Institute, National Institutes of Health, Bethesda, MD 20892, USA

**Keywords:** Bietti crystalline dystrophy, *Cyp4v3*, mouse model, light-induced injury, high-fat diet

## Abstract

Bietti crystalline corneo-retinal dystrophy (BCD) is an autosomal recessive inherited retinal dystrophy characterized by multiple shimmering yellow-white deposits in the posterior pole of the retina in association with atrophy of the retinal pigment epithelium (RPE), pigment clumps, and choroidal atrophy and sclerosis. Blindness and severe visual damage are common in late-stage BCD patients. We generated a *Cyp4v3* knockout mouse model to investigate the pathogenesis of BCD. This model exhibits decreased RPE numbers and signs of inflammation response in the retina. Rod photoreceptors were vulnerable to light-induced injury, showing increased deposits through fundoscopy, a decrease in thickness and a loss of cells in the ONL, and the degeneration of rod photoreceptors. These results suggest that an inflammatory response might be an integral part of the pathophysiology of BCD, suggesting that it might be reasonable for BCD patients to avoid strong light, and the results provide a useful model for evaluating the effects of therapeutic approaches.

## 1. Introduction

Bietti crystalline dystrophy (BCD), first reported by Gian Battista Bietti in 1937 [1], is an autosomal recessive inherited retinal dystrophy characterized by the presence of multiple shimmering yellow-white deposits in the posterior pole of the retina in association with atrophy of the retinal pigment epithelium (RPE) and choroid retinal atrophy, frequently combined with glittering crystals located at the corneal limbus [2]. The presence of the crystals is variable, although they can appear early in life, and are often seen in lymphocytes and skin fibroblasts, consistent with BCD representing a generalized metabolic disease. Although BCD is rare worldwide, it tends to be common in East Asian populations, especially in China and Japan [3], with perhaps an overall frequency of 0.5% in China. BCD is a progressive choroidal retinal dystrophy in which symptoms appear slowly and painlessly. It typically presents between the second and fourth decades of life with progressive night blindness, reduced vision, and eventually visual field constriction, often with legal blindness in the fifth or sixth decades of life. To date, no clinical treatment for this disease has been developed, although there have been some suggestive results with gene therapies in experimental mouse models [4,5,6,7].

In 2000, Jiao et al. [8] used genetic linkage analysis to localize the responsible gene to human chromosome 4q35-qter, and in 2004, Li et al. [9] identified causative mutations in the *CYP4V2* gene, which encodes a 525 amino acid protein member of the cytochrome P450 (family 4, subfamily IV, polypeptide 2), involved in fatty acid metabolism. Since then, studies have identified multiple *CYP4V2* mutations causing BCD [2,10,11,12,13,14,15,16]. The *Cyp4v3* gene in mice is highly homologous in human *CYP4V2*. *CYP4V2* is a microsomal enzyme with ω-hydroxylase activity on both saturated and polyunsaturated medium and long-chain fatty acids [17,18,19] and is expressed in almost all body tissues, especially in the RPE and neural retina, in which alterations in lipid metabolism are proposed to be the major pathogenic mechanism of BCD. An induced pluripotent stem cell model [20] and three different murine knockout models have been generated [4,5,6,7]. All of these models provide valuable insight into the disease mechanisms.

Here we describe a *Cyp4v3* KO mouse model and characterize the effects on the retina and visual function, as well as perform a long-term study on the progression of these effects both naturally and under stressed conditions. These mice models show an inflammatory response but minimal other changes in the retina under normal conditions and with a high-fat diet but do show a loss of RPE in the mice over two years old and a degeneration in the rod photoreceptors when they are subjected to stress under white light, suggesting that these conditions might be optimal for the use of this system in preclinical therapeutic trials.

## 2. Results

### 2.1. Generation of Cyp4v3 Knockout Mice

**Figure 1:**
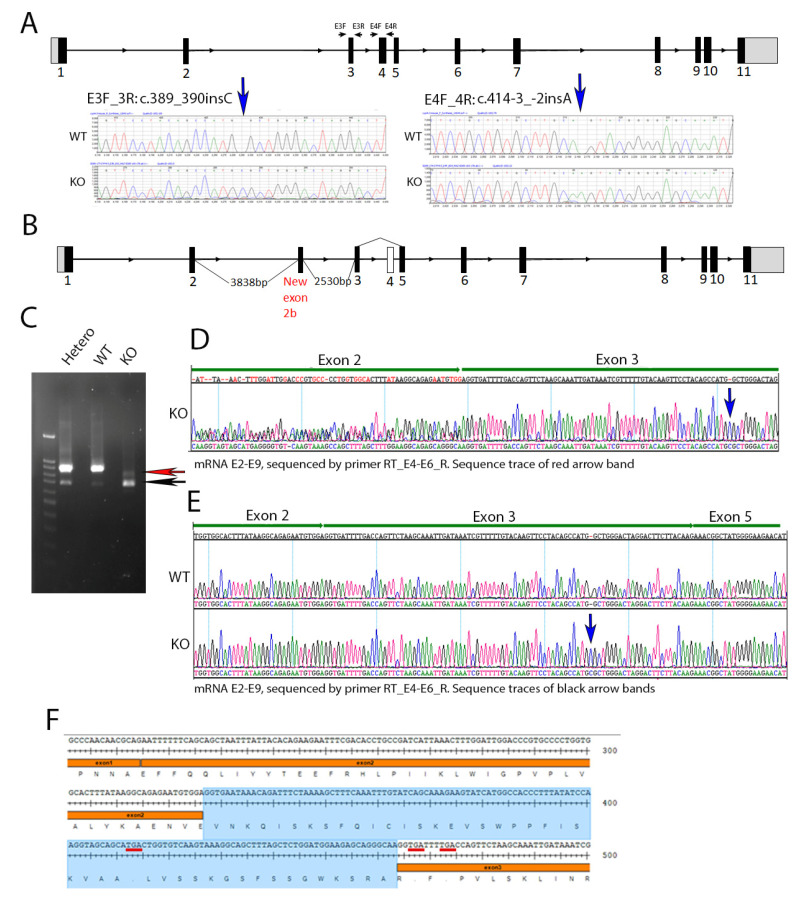
*Cyp4v3* gene structure, mutations, and expression in the KO mouse model. (**A**). Schematic of the mouse *Cyp4v3* gene showing primers used for amplification and sequence tracing, as shown for the two insertions induced in the *Cyp4v3* gene. (**B**). Novel transcript and associated gene structure in KO mice, including an extra 137 bp exon (labeled 2b) between exons 2 and 3 and skipped exon 4. (**C**). RT-PCR of Cyp4v3 mRNA spanning exons 2 to 9. The red arrow indicates the novel transcript band, including exon 2b, with reverse sequencing results shown in (**D**), with dual sequences of exons 2 and 2b in the region marked exon 2. The black arrow indicates the band for the transcript skipping exon 4, which is present in both the KO and, to a lesser extent, the wild-type sequences, the sequence trace of which is shown in (**E**,**F**). The exact sequence for the novel transcript in the KO mice with the exon 2b sequence (shaded blue) and TGA codons (underlined in red), showing the premature stop codons in exons 2b and 3.

We generated a *Cyp4v3* knockout mouse using CRISPR/Cas9 technology. The KO mouse contained two compound insertions: NM_133969.3:c.389_390insC in exon 3 and c.414-3_-2insA immediately preceding exon 4, which were identified in the *Cyp4v3* KO mouse after sequencing all 11 exons (Figure 1A). The c.389_390insC change is predicted to lead to a frameshift with at termination 37 amino acids downstream (p.(Trp130CysfsTer37)), and the c.414-3_-2insA change is predicted to decrease splice recognition at the 5′ end of exon 4 (Figure 1). To confirm the effect of these sequence changes in creating a successful knockout of the *Cyp4v3* alleles, we carried out an RT-PCR of the Cyp4v2 mRNA using multiple pairs of primers spanning the different exons of the *Cyp4v3* gene (Table S1). The combined PCR and sequencing results showed that the KO mouse *Cyp4v3* gene underwent two forms of aberrant splicing (Figure 1B). All transcripts skipped exon 4, and approximately 10% of the transcripts included an additional exon between exons 2 and 3, here termed exon 2b. Figure 1C shows the result of the RT-PCR for the wild-type, heterozygous, and knockout mice. The complete *Cyp4v3* mRNA sequence spanning Exon 2 to 9 (924 bp) was present in both the wild-type and heterozygous mice, but absent in the knockout mice, which was confirmed in the RNASeq results (see below and Figure S1). In addition to the normal mRNA transcript, another transcript skipping exon 4 (733 bp) was detected in the increasing numbers of WT, heterozygous, and homozygous KO mice (black arrow in Figure 1C), where it made up most of the transcripts. The skipping of exon 4 in some wild-type mice suggests that this is a weak splice site, which is confirmed by the inability of the GENSCAN Web Server at MIT (http://hollywood.mit.edu/GENSCAN.html) to identify it as a splice site, consistent with the complete absence of exon 4 in the c.414-3_-2insA mutant. Figure 1E shows the result of the sequencing of this band, confirming the absence of exon 4. However, in KO mice, there was a novel transcript (red arrow in Figure 1C) which contained an extra exon 137 bp in length between exon 2 and 3 and skipped exon 4 (Figure 1B,D,F). The sequencing results of the E2–E9 amplicon confirmed the absence of exon 4 (Figure S1). Thus, the combination of the two sequence changes in the KO mice resulted in premature stop codons occurring in a combination of exons 2b or 6 for all the transcript variants, giving p.(Glu109_Val110ins28*29) for those transcripts with exon 2b included, p.(W130CysfsTer34 for those transcripts without exon 2b or exon 4, and p.(Trp130CysfsTer37) for any possible transcripts without exon 2b and with exon 4 included (although this transcript is not apparent in RT-PCR or sequencing) so that the *Cyp4v3* gene contained frameshifts with premature stop codons for all possible transcripts in this animal model.

### 2.2. Phenotypic Analysis of the KO Mouse Model

**Figure 2:**
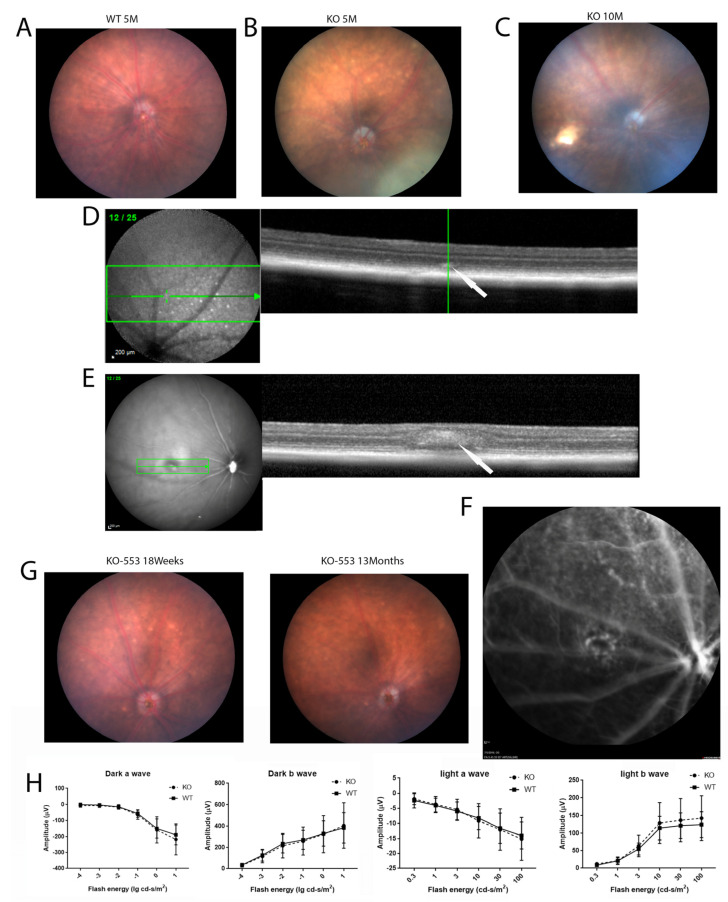
Fundus photography, OCT, and ERG of wild-type and Cyp4v2 KO mice. (**A**). Fundus images and OCT of retina in 5-month-old wild-type mouse. (**B**). Representative fundus image and OCT of 5-month-old KO mouse. (**C**). Representative fundus image of 10-month-old KO mouse, showing a retinal scar consistent with BCD. (**D**). OCT image of the KO mouse shown in (**B**) demonstrates hyperreflective spots in IS/OS of the photoreceptors in knock-out mice, as indicated by the arrow (**E**). OCT image of the KO mouse from (**C**) shows a retinal scar, as indicated by the arrow (**F**). Fluorescent angiogram of the mice shown in (**C**) and (**E**) demonstrates possible choroidal neovascularization below the scar. (**G**) Representative fundus images showing retinal crystals becoming less prominent with age. (**H**) Scotopic and photopic ERGs of 13-month-old knockout mice (N = 14) compared to wild type (N = 16).

Fundus imaging of the knockout mice was performed as the crystalline deposits are a typical characteristic of BCD. In the *Cyp4v3* KO mice, the crystals were typically visible on the retinal exam at 4 or 5 months of age (Figure 2B), and the number of crystals usually increased with age. Interestingly, in some mice, the crystals became less obvious with old age, such as mouse 553 in Figure 2G. Infrared fundoscopy-guided SD-OCT helped to display the crystals in the retina both topographically and tomographically (Figure 2D,E). SD-OCT helped to define the position of the crystals in the retina, as is shown in Figure 2D, which shows a hyper-reflective spot at the IS/OS boundary in the retina of a *Cyp4v3* KO mouse and probably represents a lipid crystal corresponding to that shown in the funduscopic view in Figure 2B. However, many of the hyper-reflective spots seen via SD-OCT did not correspond to crystals as they occurred in the INL and probably correspond to glia (Figure 2E). The phenotypes in the *Cyp4v3* KO mice varied widely, with no remarkable signs of degeneration in the retina and choroid observed, although suspected choroidal neovascularization associated with retinal scarring was rarely detected (Figure 2C,E,F). Overall, only 2 of the 91 knockout mice (2.2%) showed obvious signs of retinal degeneration (Figure 2C). In addition, the scotopic and photopic ERG results of the KO mice are essentially similar to those of WT mice. There is no significant decrease in the ERGs of the knockout mice (N = 14) compared to the wild-type (N = 16) throughout at least 13 months of age (Figure 2H).

### 2.3. Histological Analysis and Oil Red O Stain

**Figure 3:**
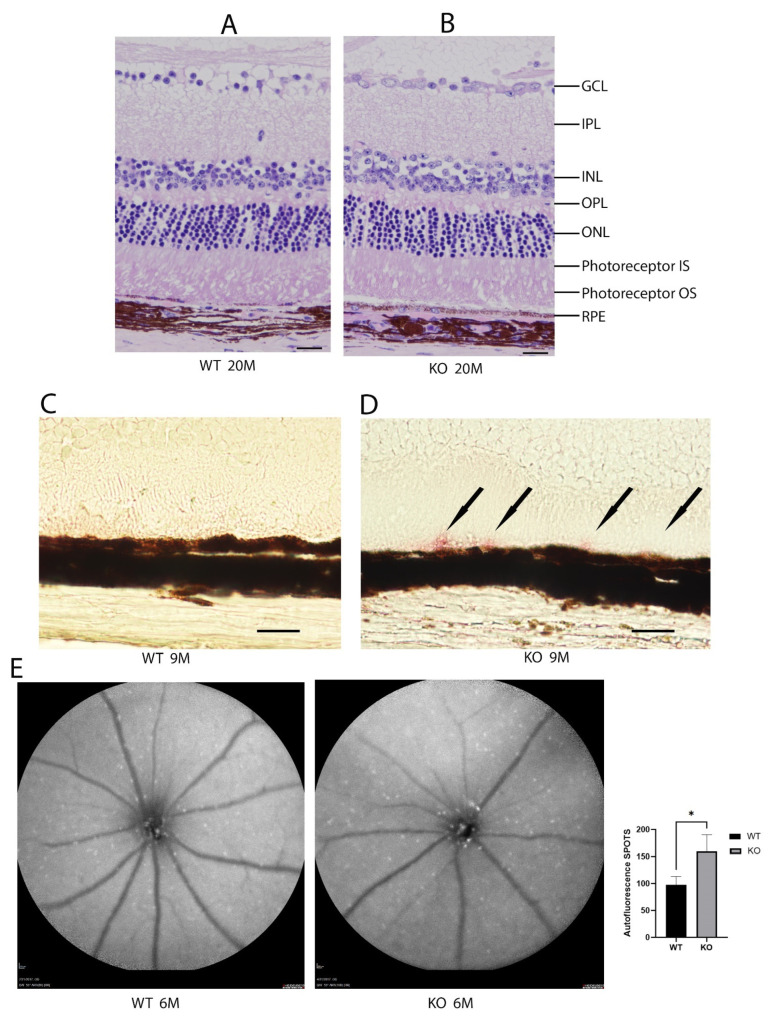
Histological and autofluorescence images of WT and KO mice. Hematoxylin and eosin staining of the retinas from 20-month-old mice (**A**). Wild-type and (**B**). KO mice. Representative Oil Red O staining images of 9-month-old mice (**C**). Wild-type and (**D**). KO mice. Oil Red O staining in the KO mice is indicated by black arrows. (**E**). Autofluorescence images of 6-month-old wild-type and KO mice and graphical representation of autofluorescence (n = 7 WT, 10 KO). Scale bar: 20 µm. * *p* < 0.05 unpaired two-tailed *t*-test.

The histologic sections from the wild-type and *Cyp4v3* KO mice show the overall preservation of the retinal structures and thickness through H&E staining for at least 20 months of age (Figure 3A,B). The Oil Red O staining of the retinal sections (Figure 3C,D) show lipid droplets in the subretinal space between the outer segments and RPE cells of 9-month-old KO mice compared with the age-matched wild-type mice. However, no excess lipid staining was present in the other retinal layers. Finally, no obvious abnormalities in the photoreceptors were seen using immunofluorescence staining for rhodopsin, cone arrestin, S-Opsin, and M-Opsin.

### 2.4. Lipid Analysis of Cyp4v2 KO Mice

No significant differences were observed between the wild-type and *Cyp4v3* KO mice when retinal and serum fatty acids were examined (Figure S2). Similarly, there were no statistically significant differences in retinal phosphatidyl choline, ethanolamine, or serine upon tandem MS analysis.

### 2.5. Decreased RPE Cell Number and Increased Autofluorescent Spots in Cyp4v3 KO Retinas

**Figure 4:**
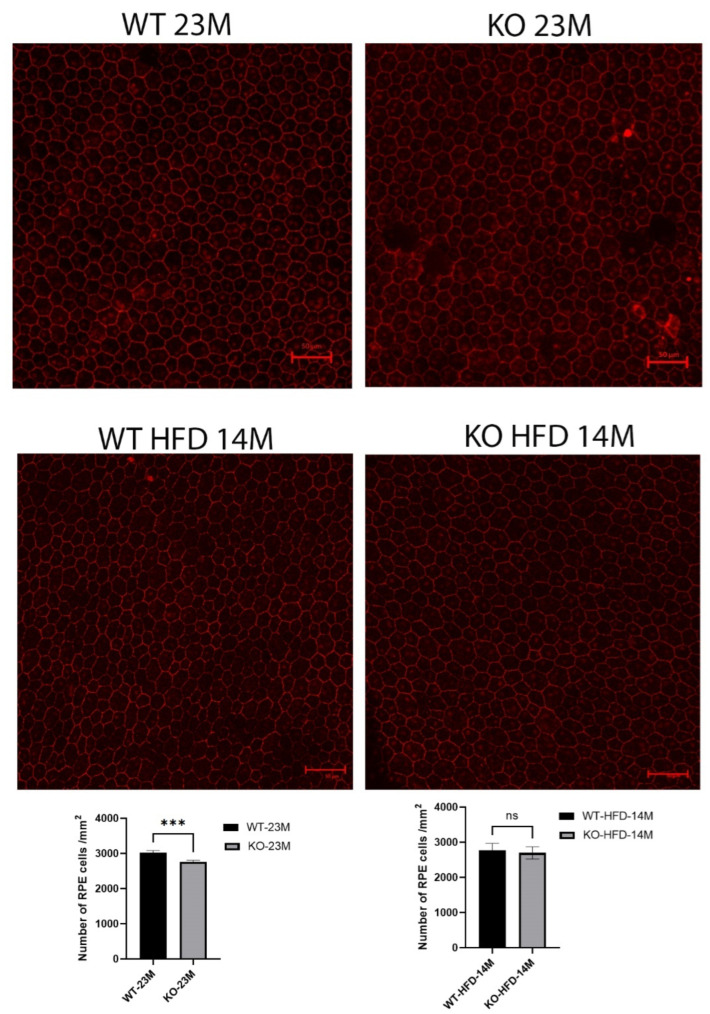
RPE-choroid flat-mount confocal immunofluorescence images with antibody to ZO-1 in 8 KO and 10 WT 23-month-old mice fed a regular diet and 5 KO and 5 WT 14-month-old mice fed the high-fat diet (because of deteriorating health beyond that age). The number of RPEs in the knockout mice was reduced slightly but with statistical significance (*p* = 0.0003) compared to the same-age wild-type mice, while no difference was seen in the mice fed with a high-fat diet. Scale bar = 50 μm, ***: *p* < 0.001, ns: not significant.

We counted the central RPE cells located within three optic disc lengths from the optic nerve for the whole of the RPE flat-mount images aligned on the optic nerve in 23-month-old mice (Figure 4). The number of RPE cells in the knockout mice was reduced slightly but with statistical significance (WT 3026 ± 53 vs. KO 2767 ± 45, *p* = 0.0003) when compared with the same-age wild-type mice.

The autofluorescent imaging of the retina, quantifying the number of fluorescent spots per image, showed that the mean number of autofluorescence spots increased in the 6-month-old knockout mice when compared to control mice (160 ± 31 vs. 97 ± 16, *p* = 0.035, Figure 3E). While some of the spots are crystalline deposits, most of them are related to clusters of inflammatory cells comprising a gliotic response. There are many more spots seen on autofluorescence than crystals in fundoscopy, and the autofluorescent spots tend to align with blood vessels, while the crystals, which are neither hyper- nor hypoautofluorescent, do not. In the *Cyp4v3* KO mouse model, there are no areas showing RPE loss, such that an increase in autofluorescent spots correlates with an increase in immune infiltration, suggesting this might be in response to an inflammatory state.

### 2.6. Inflammatory Reaction in Cyp4v3 KO Mice

**Figure 5:**
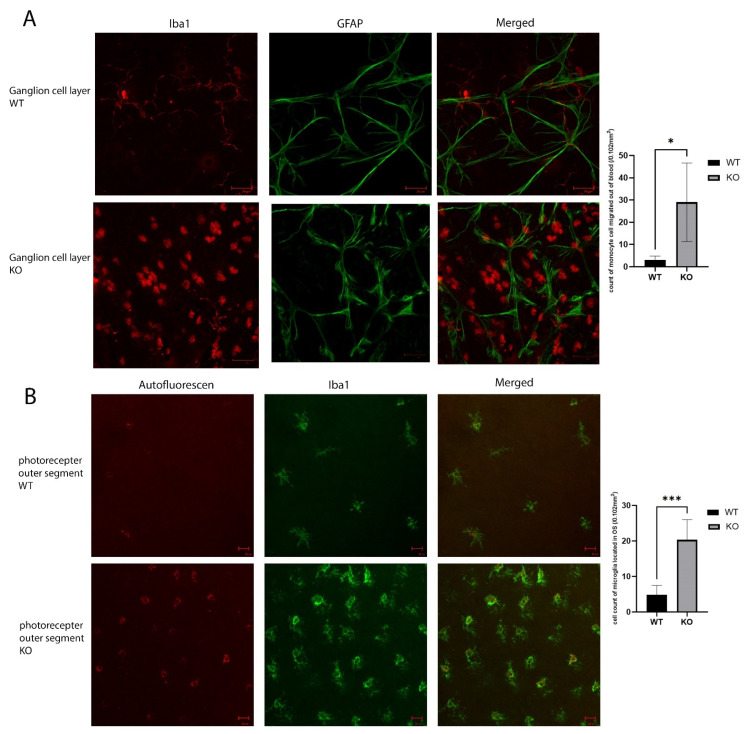
Inflammatory reaction in the KO vs. WT mice. (**A**). Immunolabeling of retinal astrocyte processes for GFAP and microglia for Iba1 in the ganglion cell layer of the retinal flat mounts showed infiltration by monocytes (the round Iba1 positive cells) in the ganglion cell layer. (**B**). Iba1 immunolabeling shows increased activated microglia cells in the photoreceptor outer segments layer. Scale bar: 20 µm. 10 WT and 10 KO mice were analyzed for each study. * *p* < 0.05, *** *p* < 0.001.

To verify the activation of an inflammatory response in the *Cyp4v3* KO mice, we performed flat-mount-immunofluorescence staining of the wild-type and KO mouse retinas. The ganglion cell layers of the KO mouse retinas showed infiltration by monocytes, seen in Figure 5A as the round Iba1 positive cells. There was only a minimal presence of monocytes in the retinal flat mounts of the control mice. In addition, there is a significant increase in microglia in the photoreceptor outer segment layer, and the microglia in the *Cyp4v3* KO mice were activated, as is shown by their increased cell volume (Figure 5B). In addition, they show increased autofluorescence, consistent with increased phagosomal activity.

**Figure 6:**
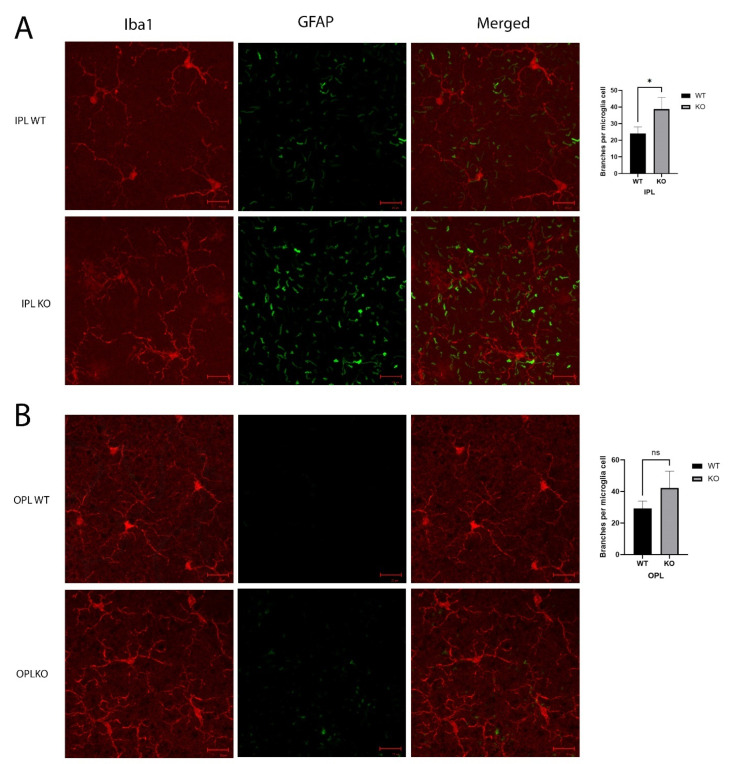
Morphology of microglia in the (**A**). IPL and (**B**). OPL. Microglia are shown by Iba1 in red and extended astrocyte processes labeled with GFAP in green. Quantification of the number of branches per microglial cell is exhibited in bar graphs. * *p* < 0.05 unpaired two-tailed *t*-test. 20 Cells were counted per mouse for 10 mice in each group. Scale bar: 20 µm. ns: not significant.

Staining the microglia in the IPL with Iba1 showed more extended and branching cell processes in the KO mice when compared to the control mice, and the astrocytes showed increased GFAP (Figure 6A). This increase in astrocytes extended to the OPL (Figure 6B), and the OPL also showed an increase in GFAP in the *Cyp4v3* KO retinas, although the overall levels were lower than those in the IPL. While the microglia exhibited a ramified morphology in both the control and *Cyp4v3* KO mice, when morphological alterations in the microglia cells, including microglial brunching and the number of processes, were quantified in the IPL and OPL (Figure 6), there was a statistically significant increase in the branches in the *Cyp4v3* KO mice compared to the control mice in the IPL (38 ± 7 to 24 ± 5, *p* < 0.05), meaning a smaller suggestive increase in the OPL that is not statistically significant.

### 2.7. RNA-Seq Analysis of Cyp4v3 KO Mouse Retinas

**Figure 7:**
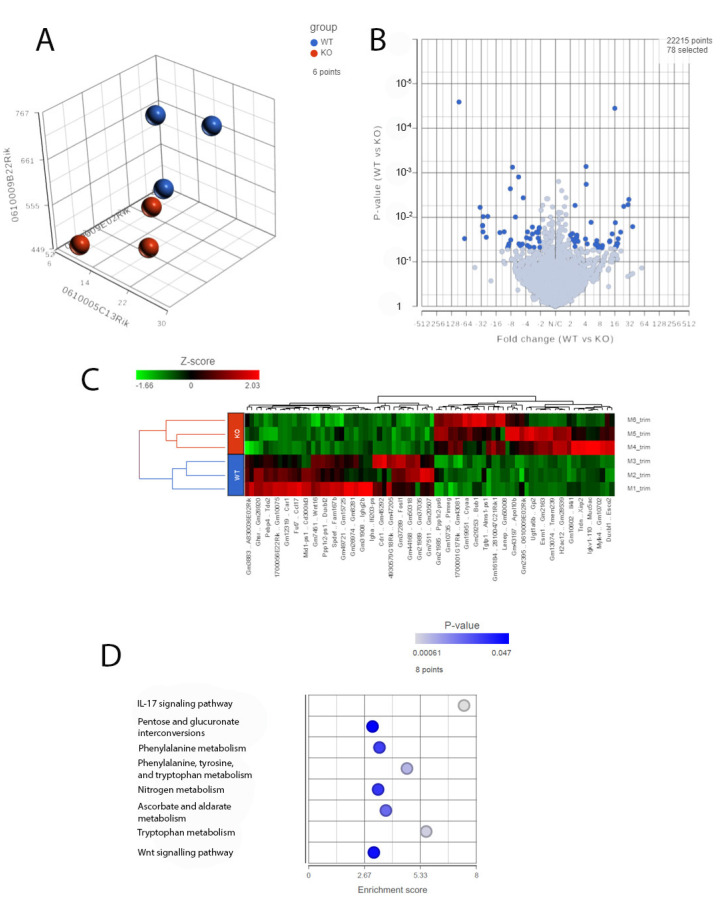
RNA-Seq analysis of 1-year-old wild-type and KO mice. (**A**). PCA plots of RNA-Seq data showing the characteristics of the overall gene expression of each sample. (**B**). Volcano plot showing expression changes between the wild-type and KO mice. (**C**). Heat map displaying the expression profile of the top differentially expressed genes. (**D**). GO pathway enrichment in the KO mice compared with the wild-type mice (adjusted *p* < 0.05).

An increased inflammatory and immune response in the *Cyp4v3* KO mice was supported not only by the results of the immunofluorescent staining, but also by the RNA-Seq carried out on the 1-year-old mice. PCA of the RNA-Seq results from three mice showed clustering of the control and *Cyp4v3* KO mice (Figure 7A), although, as with the functional and morphological metrics, there is some scatter among the members of each group. A volcano plot indicating the increased and decreased expression of the genes in the *Cyp4v3* KO vs. control mouse retinas is shown in Figure 7B. A total of 78 genes showed significantly altered expression in the *Cyp4v3* KO mice: 38 increased and 40 decreased (Table S2). Figure 7C shows a heat map display of the top differentially displayed genes, and the enrichment scores for the gene ontology (GO) pathways is shown in Figure 7D. The Kyoto Encyclopedia of Genes and Genome (KEGG) pathways were also examined and showed similar results, with an increase in the elements of the IL-17 signaling pathway (*p* = 0.0151, q = 0.425) and the Wnt signaling pathway (*p* = 0.0435, q = 0.454) and decreases in phenylalanine metabolism (*p* = 0.0318, q = 0.295) and phenylalanine, tyrosine, and tryptophan biosynthesis (*p* = 0.011, q = 0.295). As mentioned above, no *Cyp4v3* transcripts, including exon 4 were identified via RNA-Seq (Figure S1B), confirming the results of the RT-PCR shown in Figure 1C.

### 2.8. High-Fat Diet Stress and Light Injury in Cyp4v3 KO Mice

Inflammatory processes were involved in the KO mice, however, no obvious functional changes to the retina were identified. In order to dissect the differences between the wild-type and *Cyp4v3* KO mouse retinas, we subjected the KO and control mice to a high-fat diet and separately to white light stress. The mice subjected to a high-fat diet did not show any obvious retinal lesions, and there were no obvious scotopic or photopic ERG differences between the KO and wild-type mice at 14 months (Figure S3). The mice could not be studied beyond this point because their general health began to deteriorate. Thus, in this mouse model, HFD is associated with profound systemic alterations but no obvious changes in ocular outcomes.

**Figure 8:**
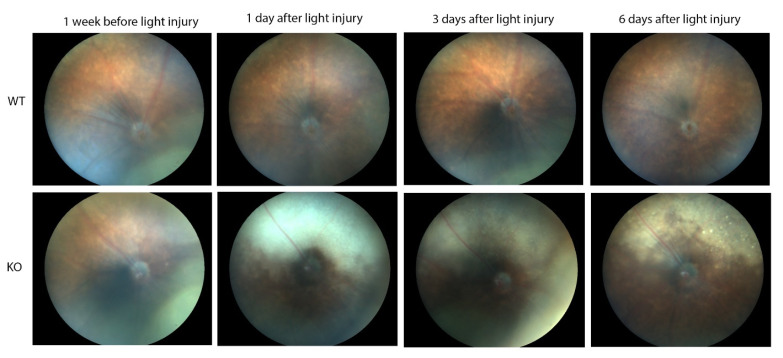
Fundus photographs showing light-induced injury in 17-month-old WT (10) and KO (6) mice. The time points are one week before light stress and one, three, and six days after light exposure.

**Figure 9:**
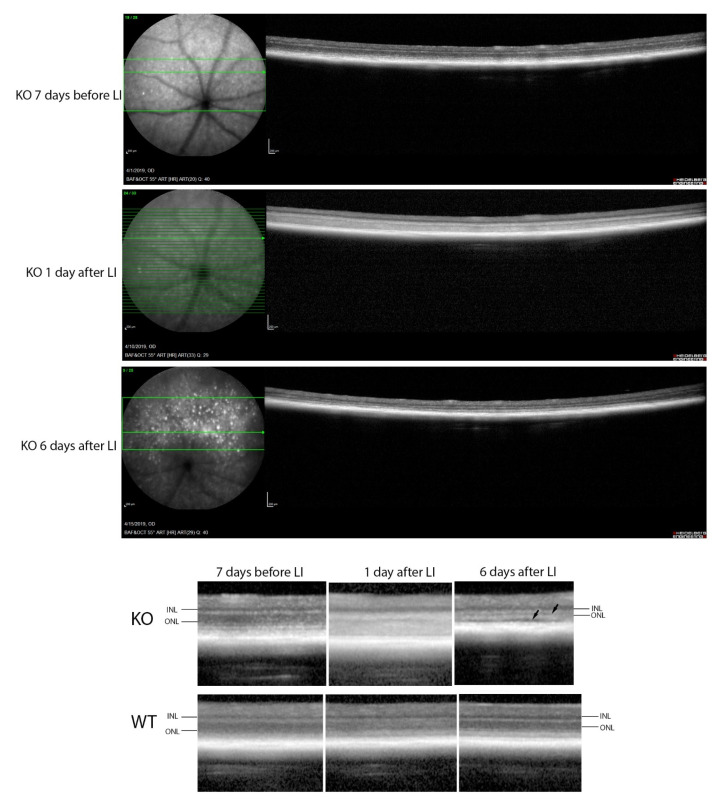
OCT imaging of mice before and after light stress. **Top**: KO mouse retina 7 days before and 1 and 6 days after light stress. **Bottom**: comparison of WT and KO retinas at the same time points, with arrows showing a narrowing of the ONL, and separation of the photoreceptor layer from the RPE in the KO but not the WT. Scale bar = 200 μm.

**Figure 10:**
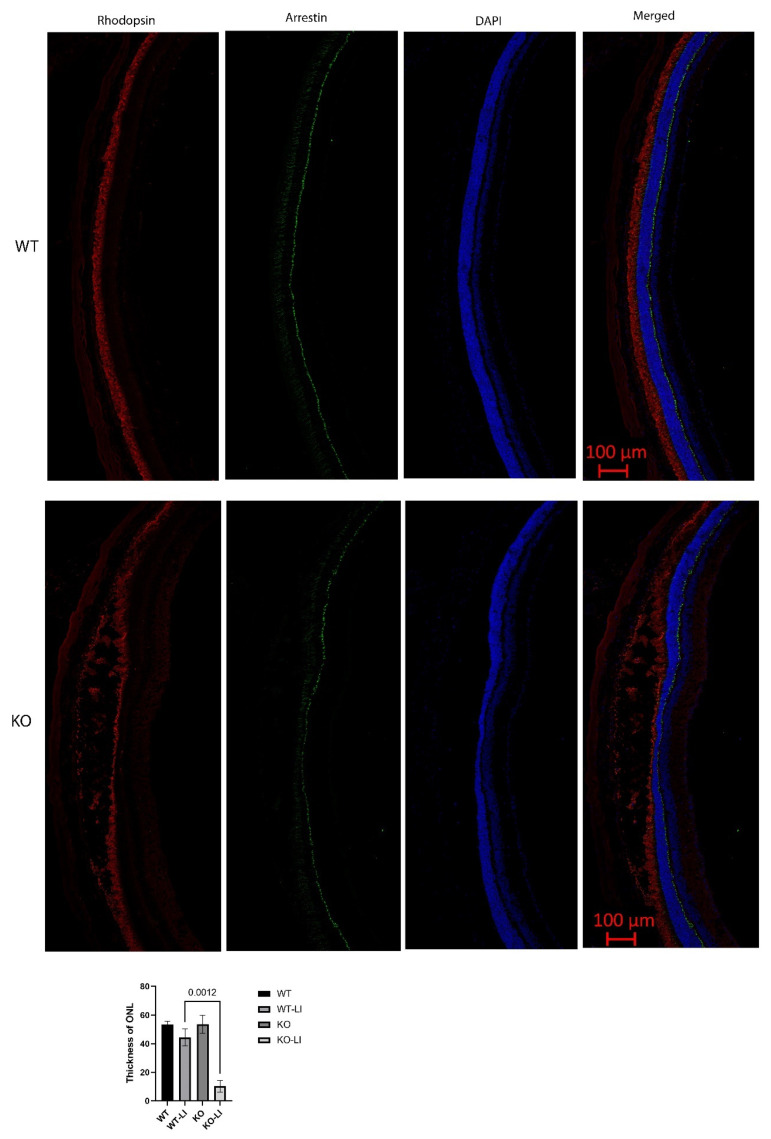
Immunofluorescence staining of retinal sections 7 days after light-induced injury. Rod photoreceptors, marked with rhodopsin (red), were reduced, damaged, and in disarray. Cone photoreceptors, marked with arrestin (green), remained largely intact. Quantification of ONL thicknesses of 6 KO and 10 WT mice shows a statistically significant decrease in the KO as compared to the WT in the dorsal retina, which is the region exposed to light, seen in the center of the retinal sections. LI: light injured.

In contrast, major differences were seen between the wild-type and *Cyp4v3* KO mice when the effects of light injury were analyzed using fundus photographs, in vivo OCT imaging, and histology. Light exposure in the wild-type animals usually requires exposure for a more prolonged time, and acts through oxidative damage and inflammation, proceeding through an edematous response that resolves when followed by retinal degeneration, characterized by photoreceptor loss with apoptotic and necroptotic changes [21]. Fundus photographs in the Cyp4v2 KO mice after 1 hour of light exposure show large amounts of edema in the dorsal retina one day after light injury in 17-month-old KO mice when compared with same wild-type littermates (Figure 8). While the edema begins to resolve at 3 and 6 days after light exposure, there is a residual separation of the RPE from the photoreceptor layer in anticipation of later scarring and an increased number of flecks in the light-exposed dorsal retina as compared to both the wild-type mouse retina and the less-damaged ventral retina. These changes are also confirmed by OCT imaging (Figure 9), which shows the edematous changes 1 day after light exposure followed by a narrowing of the ONL, less distinct inner and outer segments of the photoreceptor layers, and some separation of the RPE from the photoreceptor layer at 6 days after exposure. Immunofluorescence staining of the retinal sections 7 days after light injury showed a large loss and degeneration of rod photoreceptors in the light exposed dorsal retina but with relative preservation of the cone photoreceptors (Figure 10). The quantification of ONL thicknesses in the light-injured area showed a statistically significant difference between the wild-type and KO mice after light injury, with major changes once more seen in the light-injured area shown in the center of the section. These results are also confirmed by the H&E-stained histological sections of the WT and KO retinas 7 days after light exposure (Figure S4). Once more, the light damage occurs in the dorsal retina, and is characterized by the death and disarray of the photoreceptor IS and OS layers accompanied by decreased thickness and cell numbers in the ONL and separation of the RPE from the photoreceptor layer.

## 3. Discussion

A number of BCD mouse models have been established using different gene editing strategies [4,5,6,7], each showing somewhat different phenotypes and responses to stress. Here we describe a *Cyp4v3* KO mouse model with an inflammatory reaction in the retina, a reduced RPE cell number, and photoreceptors that are vulnerable to white light exposure. This model recapitulates some characteristic features of BCD in human patients, although the phenotype is milder than that seen in humans. In addition, in humans, fundus autofluorescence (FAF) is not helpful in assessing the evolution of crystalline deposits because these dots are neither hypoAF nor hyperAF in nature [22]. Thus, the peripheral retina shows normal FAF as long as there is no damage. However, in BCD, the posterior pole and the paracentral retina develop hypoAF and clearly defined confluent-patched areas corresponding to local RPE loss sections. Thus, some differences in phenotype might be related to different diagnostic modalities available in the two species, the different lifespans in humans and mice allowing for the more complete development of the disease phenotype in humans, or perhaps to other genetic differences in mice and humans.

As well as differing in severity from human BCD, the various mice models themselves show differing, though overlapping, phenotypes. In our model, there is a slight decrease in RPE cell number, and the retinal crystals are deposited beginning at 4–5 months of age. This is similar to the models described by Lockhart et al. [7] and Jia [6], but later than that, described by Wang et al. [5], while the mouse model described by Qu et al. did not show significant changes without stress [4]. In addition, we did not detect any obvious abnormalities in the photoreceptors by immunofluorescence staining, which differs from the mouse model reported by Wang Y et al. [5], although our model shows similar inflammatory changes. Although the crystals in our model are phenotypically similar to the yellow spots seen in the Crb1^rd8^ mouse retina [23], the mice used in this model did not contain the Crb1^rd8^ mutation. While our model did not show ERG changes, the models described by Wang and Jia both showed decreases in the scotopic and photopic a- and b- waves by 6 months. Our mouse model behaved similarly to control mice when fed a high-fat diet, in contrast to that of Qu et al. [4], which showed changes in fundus photography increased autofluorescence with decreased ERG amplitudes. In contrast, when challenged with a single challenge with white light, our mouse model displayed a typical inflammatory response with edema followed by damage to rod photoreceptors and increased retinal deposits, similar to the results with the model described by Wang et al. [5]. While the experimental light exposure should apply equally to both the dorsal and ventral retina, the worst damage was consistently localized to the dorsal retina. While the reason for this is unclear, it might relate to differences in the distribution of cell types in the dorsal and ventral retina, which have been correlated with patterns of retinal diseases [24,25,26]. Alternatively, it might be caused by a higher exposure of the dorsal than ventral mouse retina to light over time as mice tend to focus on close objects on the ground, or even the tendencies of mice to squint during the later parts of the light exposure, which could decrease the exposure of the ventral retina. Most of the structural changes noted in the *Cyp4v3* KO mice were focal, which would not be revealed with a functional assay such as a full-field ERG. On the other hand, these defects are consistent with the retina of KO mice being more vulnerable to oxidative and inflammatory insults, consistent with the results above. In addition, while there were some small apparent differences in the fatty acid and lipid profiles in the retina and serum of our mouse model, they were variable and not statistically significant. In contrast, the models described by both Qu et al. [4] (decreases in C22:6, C18:3, C18:2, and C18:1) and Wang et al. (increases in C20:3N3, C20:3N6, C20:4N6, C22:0, C22:5N3, and C22:5N6) [5] both showed medium chain fatty acids levels, although they were somewhat different in each model. Once more, differences in phenotypes among the mice models might relate to variations in the experimental approaches and husbandry but seem more likely to relate to differences in the genetic backgrounds of the mice, which are likely to have been maintained in separate colonies for long periods of time, even though they are theoretically the same strain.

Because of the known function of CYP4V2 as a selective omega-hydroxylase of both saturated and polyunsaturated medium-chain fatty acids in humans [17,18], attention on both human and mice models has centered on the role of fatty acid metabolism in the pathogenesis of BCD. Abnormal serum fatty acid compositions in human BCD patients have also varied in different clinical studies [19,27]. Lai et al. found increased C18:0 and correspondingly lower C18:1N9 and lower total monounsaturated fatty acid levels. Lee et al. found lower conversion of FA precursors into N3 polyunsaturated fatty acids consistent with a deficiency in fatty acid desaturation. In a cultured human RPE cell model of BCD, Hata et al. induced pluripotent stem cells from individuals who carried specific *CYP4V2* mutations to generate RPE cells [20]. These cells showed vacuolated cytoplasm similar to the degenerative changes observed in individuals with BCD. In addition, the cells accumulated glucosylceramide and free cholesterol in association with lysosomal malfunction and decay of autophagic flux, involving severe impairment and cellular apoptosis. These could all be rescued by reducing free cholesterol.

Our *Cyp4v3* KO mouse model shows strong evidence of an inflammatory reaction in the retina, including migration of monocytes from the bloodstream into the ganglion cell layer, increased GFAP expression in the astrocytes and Müller cells, increased activated microglia cells in the outer photoreceptor segment, and RNA-seq data supporting an immune response. In this regard, it is noteworthy that mRNA levels of Cryaa, a small heat shock and stress protein in the RPE [28], are increased by over 7-fold in the Cyp4V2 KO mouse retina (Table S2). Imbalanced lipid metabolism represents a logical candidate for the cause of these inflammatory changes. Zhang et al. used BCD patient-derived iPSCs- derived RPE cells to show that *CYP4V2* mutations disrupted fatty acid homeostasis, resulting in an accumulation of PUFAs [29]. The increased levels of PUFAs increased the mitochondrial generation of reactive oxygen species, activating p53-independent apoptosis. This process could induce an inflammatory response in the intact retina. Similarly, some arachidonic acid metabolites and omega-3 essential fatty acids (EPA and DHA) act as autocoids to regulate immune responses, and PUFAs are the precursors of maresin and resolvin, which possess anti-inflammatory and immunoregulatory properties [30]. Taken together, this suggests that defective Cyp4v3 activity may cause an imbalance in fatty acid species that not only induces an inflammatory response but also impairs its resolution.

The photoreceptors, and especially rods, in our *Cyp4v3* KO mouse model, are vulnerable to light stress. Less than two hours of exposure to intense light reduced the ONL thickness in some areas of the dorsal retina by up to 80%, with edema and degenerative changes seen in the rods. The damaged photoreceptors trigger an immune response, which is probably meant to clear the debris, but when it fails, the clearance mechanisms are overwhelmed, resulting in chronic damage and accumulation of lipid crystals. While the changes in the unstressed *Cyp4v3* KO model are relatively minor and difficult to quantitate, such that they do not provide a reasonable metric to monitor disease progression or response to therapy. In contrast, when combined with light exposure that leaves control mice essentially intact, the phenotypic changes are significant enough to provide a serviceable metric to follow a therapeutic response.

Overall, these results strongly suggest that, in the absence of *Cyp4v3,* the retina undergoes oxidative damage with a resultant inflammatory response, and this process is exacerbated by light exposure. Not only does this model provide a test system for possible therapies for BCD, but these results suggest that avoiding light exposure might be a therapeutic adjunct to slow progression in BCD patients. However, the exact pathogenic mechanisms involved in RPE malfunction and in the subsequent damage to photoreceptors in humans still need further investigation.

## 4. Materials and Methods

### 4.1. Animals

C57BL/6 mice used in this study were cared for in accordance with the recommendations of the Guide for the Care and Use of Laboratory Animals, Institute of Laboratory Animal Resources, the Public Health Service Policy on Humane Care and Use of Laboratory Animals, approved by the Animal Care and Use Committee (ACUC) of the National Eye Institute as approved in NIH ASP NEI-576, and adhered to the Association for Research in Vision and Ophthalmology (ARVO) Statement on animal use in ophthalmic and vision research. The *Cyp4v3* KO mice were generated by the NEI Genome Core Facility.

### 4.2. Genomic DNA Extraction, PCR, RT-PCR, and Sequencing

Total tail DNA and retinal RNA were extracted using Qiagen kits. The quality and quantity of DNA and RNA were assessed using a NanoDrop spectrophotometer (Thermo Fisher Scientific, Waltham, MA, USA). The RNA was reverse transcribed to cDNA by Invitrogen SuperScript III reverse transcriptase kit (Invitrogen, Waltham, MA, USA). PCR products were purified and analyzed by Sanger sequencing using an ABI 3130 sequencer with Big Dye Terminator Ready reaction mix, according to the manufacturer’s instructions (Applied Biosystems, Foster City, CA, USA). Sequencing results were analyzed using Mutation Surveyor v3.30 (Soft Genetics, State College, PA, USA) or DNASTAR Navigator 17 (DNASTAR, Madison, WI, USA).

### 4.3. Color Fundus Photography (CFP), Spectralis-Domain Optical Coherence Tomography (SD-OCT), and Fundus Autofluorescence (FAF)

CFP images were adopted using a Micro III system (Phoenix Research Laboratories, Pleasanton, CA, USA). OCT and FAF were performed using a Heidelberg Spectralis platform (Heidelberg engineering, Franklin, MA, USA). The pupils of the mice were dilated with tropicamide (1%, Alcon, Ft. Worth, TX, USA) and phenylephrine (2.5%, Alcon, Ft. Worth), and then mice were anesthetized with intraperitoneal ketamine (100 mg/kg) and xylazine (6 mg/kg). Lubricant eye drops (Systane, Alcon, Ft. Worth) were applied on the corneal surface. The CFP images were viewed and captured initially, followed by SD-OCT and FAF (excitation at 488 nm). The 55° imaging data were acquired centering on the optic nerve head. For fluorescent angiography, after CFP and OCT images had been acquired, 50 µL of 10% sodium fluorescein was injected subcutaneously, and fluorescein angiography was performed using the fluorescence mode of the Heidelberg Spectralis system.

### 4.4. Electroretinographic Analysis

Electroretinographs (ERGs) were recorded using an Espion E2 system (Diagnosys, Littleton, MA, USA). Mice were dark-adapted overnight and prepared for recording in darkness under dim-red illumination. The mice were anesthetized with pupils dilated and corneal lubricated as previously described [31]. Flash ERGs recordings were obtained from electrodes placed on the corneas, with the reference electrode placed in the mouth and the ground subdermal electrode at the tail. Scotopic ERG responses were obtained at increasing light intensities over the ranges of 1 × 10^−4^ to 10 cd·s/m^2^ under dark-adapted conditions. Before the photopic ERG, light adaption was performed with a steady background illumination. Photopic ERG responses were recorded with light intensities of 0.3 to 100 cd·s/m^2^. The stimulus interval between flashes varied from 5 s at the lowest stimulus strengths to 60 s at the highest. After that, a 30 Hz flicker ERG was recorded. Five responses were averaged per result. Analysis of a-wave and b-wave amplitudes were performed. Espion ERG Data Analyzer software (v2.2) was used to digitally filter out high-frequency oscillatory potential wavelets. The a-wave amplitude was measured from the baseline to the negative peak, and the b-wave was measured from the a-wave trough to the maximum positive peak.

### 4.5. Histology

Mice were euthanized by CO_2_ inhalation, and eyeballs were harvested for cryosections or paraffin-embedded sections. The globes were fixed in 10% neutral buffered formalin (Sigma-Aldrich, St. Louis, MO, United States) overnight at room temperature, dehydrated stepwise using ethanol, and then embedded in paraffin. Five μm sections through the pupillary-optic nerve axis were prepared using a Microtome. The sections were deparaffinized, hydrated, and finally stained with Hematoxylin and Eosin (H&E). Frozen retinal sections were stained with Oil Red O solution (Abcam). Images were examined using an Olympus BX-51 microscope (Olympus, Bartlett, TN, United States).

### 4.6. Immunofluorescent Assessment of Mouse Retinas

Mouse eye cups were fixed in 4% paraformaldehyde in 1x PBS for 3 h. After stepwise dehydration using glucose, the eye cups were embedded in an Optimal Cutting Temperature (OCT) (Tissue-Tek, Torrance, CA, USA) with appropriate orientation to allow for the cutting of sagittal sections. Ten µm thickness cryosections were permeabilized with 0.3% Triton X-100 in PBS, the sections were blocked with blocking buffer (PBST, 5% BSA, 1% goat serum) for 1 h at room temperature. The sections were then incubated in a humidified chamber to prevent evaporation with primary antibodies overnight at room temperature. After three washes in PBST for 15 min each, the sections were incubated with the corresponding secondary antibodies at room temperature for 1.5 h followed by three PBST washes and finally staining with DAPI (Sigma-Aldrich, NY, NY, United States) for 30 min.

Retina and RPE-choroid complexes were dissected out for flat-mounted analysis, using a protocol similar to that for section immunofluorescence staining except for incubating in a 96-well round bottom plate. Primary antibodies used for staining were anti-rhodopsin (MAB5356, Millipore, Burlington, MA, USA, 1:500), anti-cone arrestin (AB15282, Millipore, 1:500), anti-ZO-1 (40-2200, Invitrogen, 1:200), anti-GFAP (13-0300, Invitrogen, 1:400), anti-Iba1(019-19741, wako, 1:200), anti-Glutamine synthestase (MAB302, Millipore, 1:300), anti-vimentin (5741, Cell signaling, 1:300), and anti-CD11b (MCA711, Bio-rad, 1:300). Images were collected with a confocal laser scanning microscope (Zeiss LSM 800, Carl Zeiss Inc., Thornwood, NJ, USA).

### 4.7. Retinal Light Injury (LI) and High-Fat Diet Stress

A total of 6 *Cyp4v2* KO and 10 littermate control mice were dark-adapted in a dark room for 7 d and then subjected to pupillary dilation with topical tropicamide (1%; Alcon, Ft. Worth) and phenylephrine (10%; Alcon, Ft. Worth). After full dilation, animals were placed into an aluminum-foil coated cage and exposed to 2 × 10^4^ lux of diffuse white fluorescent light (Sunlite Manufacturing) for 2 h. After light exposure, the mice were maintained in typical conditions of ambient cyclic light (~100 lux, 12:12 h) under which the animals were normally housed. High-fat diet chow with 60% kcal% fat (D12492i) was purchased from Research Diets. A total of 63 mice under high-fat diet stress were given the special feed from postnatal week 3 until 14 months old.

### 4.8. RNA-Seq

Total RNA samples were purified from the retina of 12-month-old wild-type and KO mice (n = 3) using a Qiagen kit. After passing the quality control, RNA-sequencing was carried out using an Illumina NovaSeq platform utilizing a paired-end 150 bp sequencing strategy (Novogene Corporation Inc., Sacramento, CA, USA). Analysis was performed using Partek Flow (https://partekflow.cit.nih.gov/flow) on the NIH Biowulf supercomputing cluster. 

### 4.9. Retinal and Serum Lipid Analysis

For serum collection, 9-month-old mice were anesthetized. Whole blood samples were collected from retro-orbital venous plexus, and then left them undisturbed at room temperature for 30 min. Remove the clot by centrifuging at 2000 g for 10 min. The supernatants were pipetted to a new centrifuge tube and stored at −80 freezer. Retinal tissues were dissected under a stereoscope. Retinal and serum lipid analysis were carried out as previously described [32,33]. Briefly, lipids were extracted from 100 μL of plasma or 100 μg of whole retinas following the method of Bligh and Dyer with minor modifications [32,33]. Fifty nmol each of 15:0, 17:0, and 23:0, and 4 nmol of 30:3n6 were added as internal standards. One milliliter of 16.6% concentrated HCl in methanol was added, and the tubes were sealed under N2 with Teflon-lined caps, and heated at 100 °C overnight. The tubes were cooled on ice and fatty acid methyl esters were extracted and processed as previously described [34]. All reagents for fatty acid extraction and derivatization were from Sigma-Aldrich. Fatty acid methyl esters were identified using an Agilent Technologies 7890A gas chromatograph with a 5975C inert XL mass spectrometer detector (Agilent Technologies, Lexington, MA, USA) operated in the electron impact total ion and single ion monitoring modes. The injection volume was 1 μL and the inlet was held at 280 °C and set to pulsed split less mode. An Agilent Technologies DB-23 column was used with a helium carrier gas flow rate of 1.9 mL/min. PUFAs were identified by using the *m*/*z* 79.1, 108.1, and 150.1 in single ion monitoring mode and the full scan mass spectra in total ion mode. Fatty acid methyl esters were quantified using an Agilent Technologies 6890N gas chromatograph with a flame ionization detector. Sample concentrations were determined by comparison to the internal. The injection volume was 1 μL, and the inlet was held at 280 °C and set to pulsed split mode (10:1 ratio). An Agilent Technologies DB-23 column was used with hydrogen carrier gas at a constant pressure of 13.1 psi. PC, PE, and PS molecular species were determined by high-resolution MS and MS/MS using accurate mass measurements in positive ion mode.

### 4.10. Statistical Analysis

All data were analyzed using the statistical software package GraphPad Prism Software, Version 9.2.0. For two-way comparisons of data following a Gaussian distribution, independent datasets were analyzed with an unpaired two-tailed *t*-test. Statistically significant differences between two or more groups were tested using a one-way ANOVA with a post hoc Scheffe test. A *p*-value < 0.05 was set as the basis for rejecting the null hypothesis. In all graphical representations, the error bars indicate standard deviation (SD).

## Data Availability

All relevant data are included in this manuscript.

## Supplementary Materials

The following supporting information can be downloaded at: https://www.mdpi.com/article/10.3390/ijms232113108/s1.
