# Supplementary figures and images for: A Bietti Crystalline Dystrophy Mouse Model Shows Increased Sensitivity to Light-Induced Injury
Source: Int J Mol Sci. 2022 Oct 28;23(21):13108. doi: 10.3390/ijms232113108 (PMC9658898; doi:10.3390/ijms232113108)

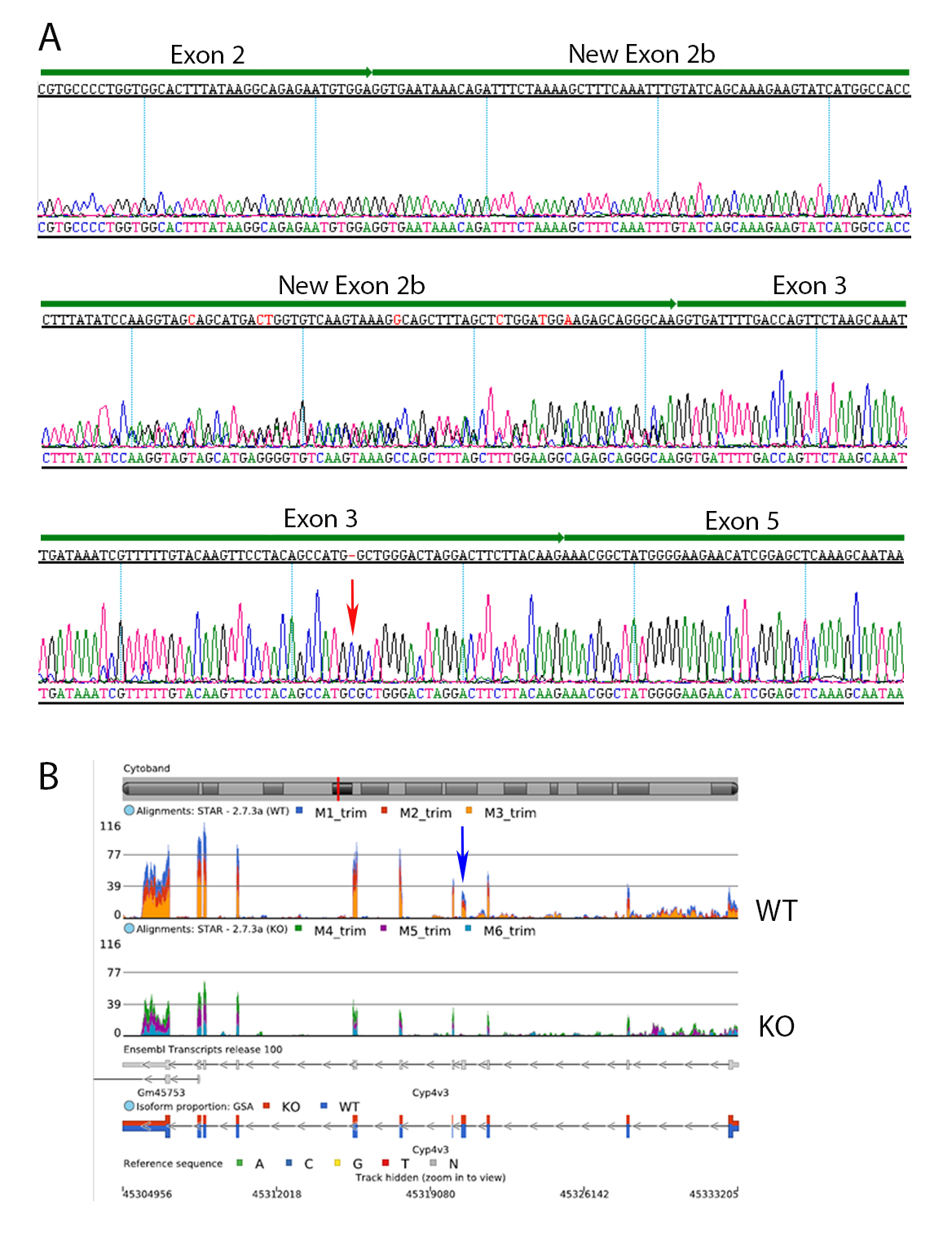

Supplement: Supplementary file 1 [file ijms-23-13108-s001.zip › figure S1.jpg]

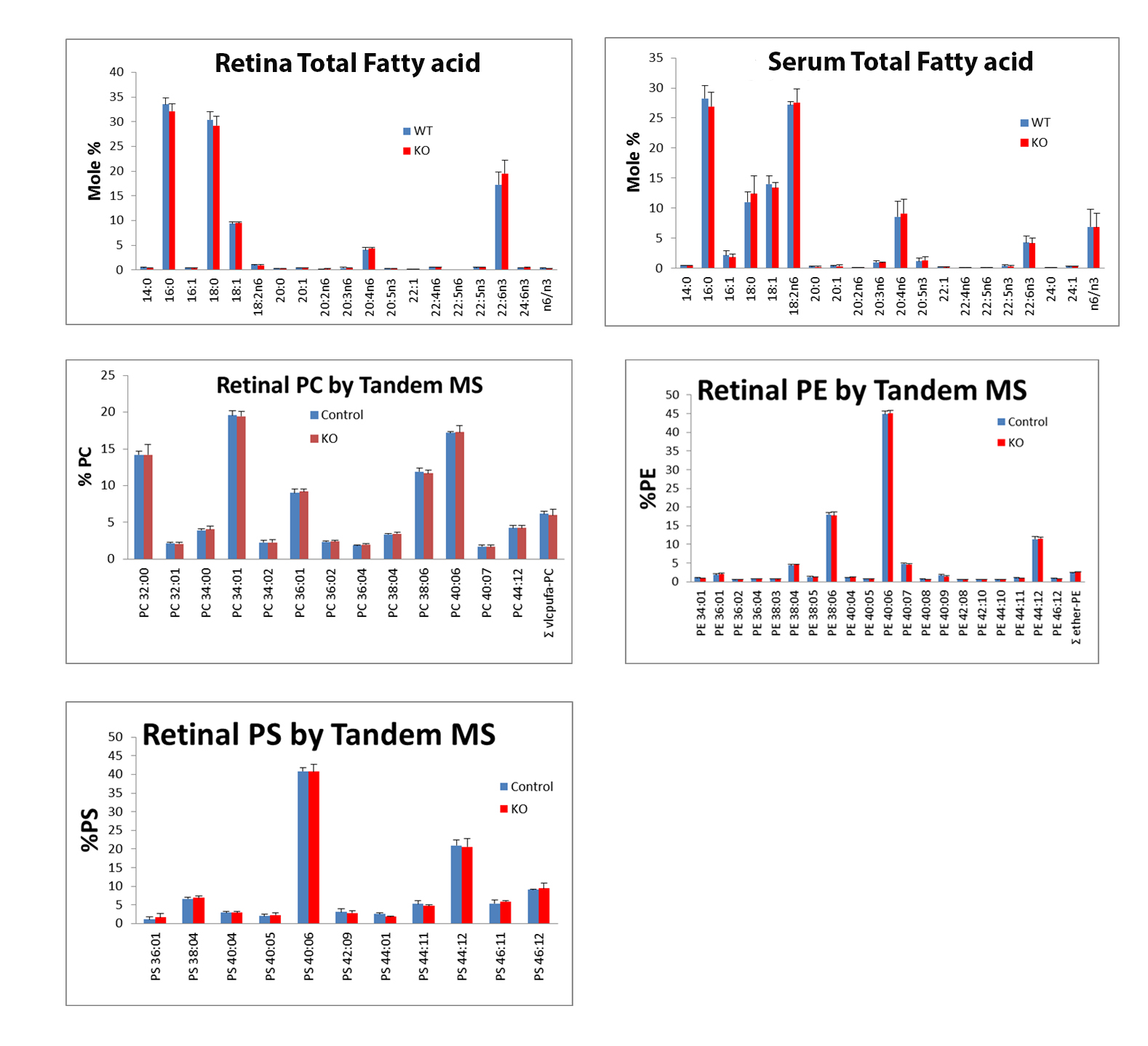

Supplement: Supplementary file 1 [file ijms-23-13108-s001.zip › figure S2.jpg]

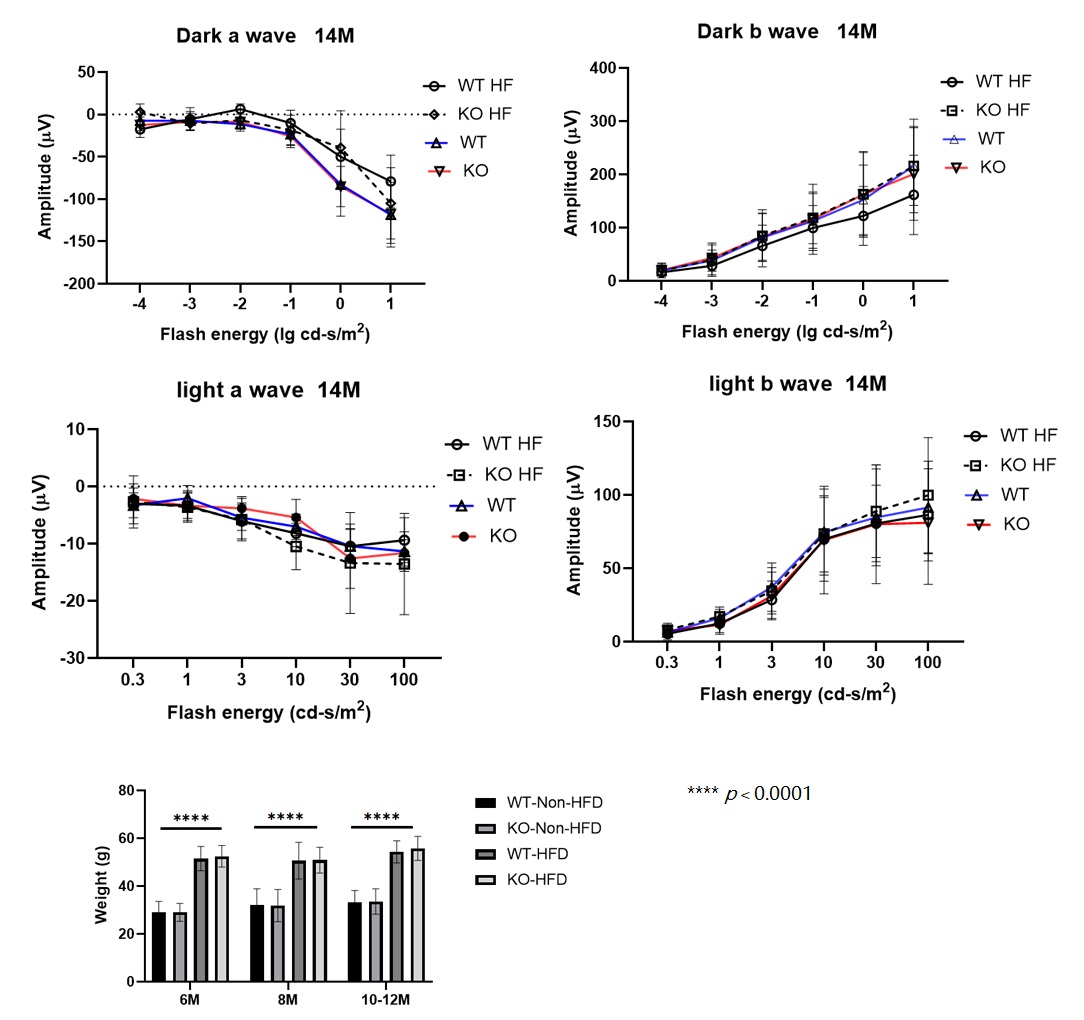

Supplement: Supplementary file 1 [file ijms-23-13108-s001.zip › figure S3.jpg]

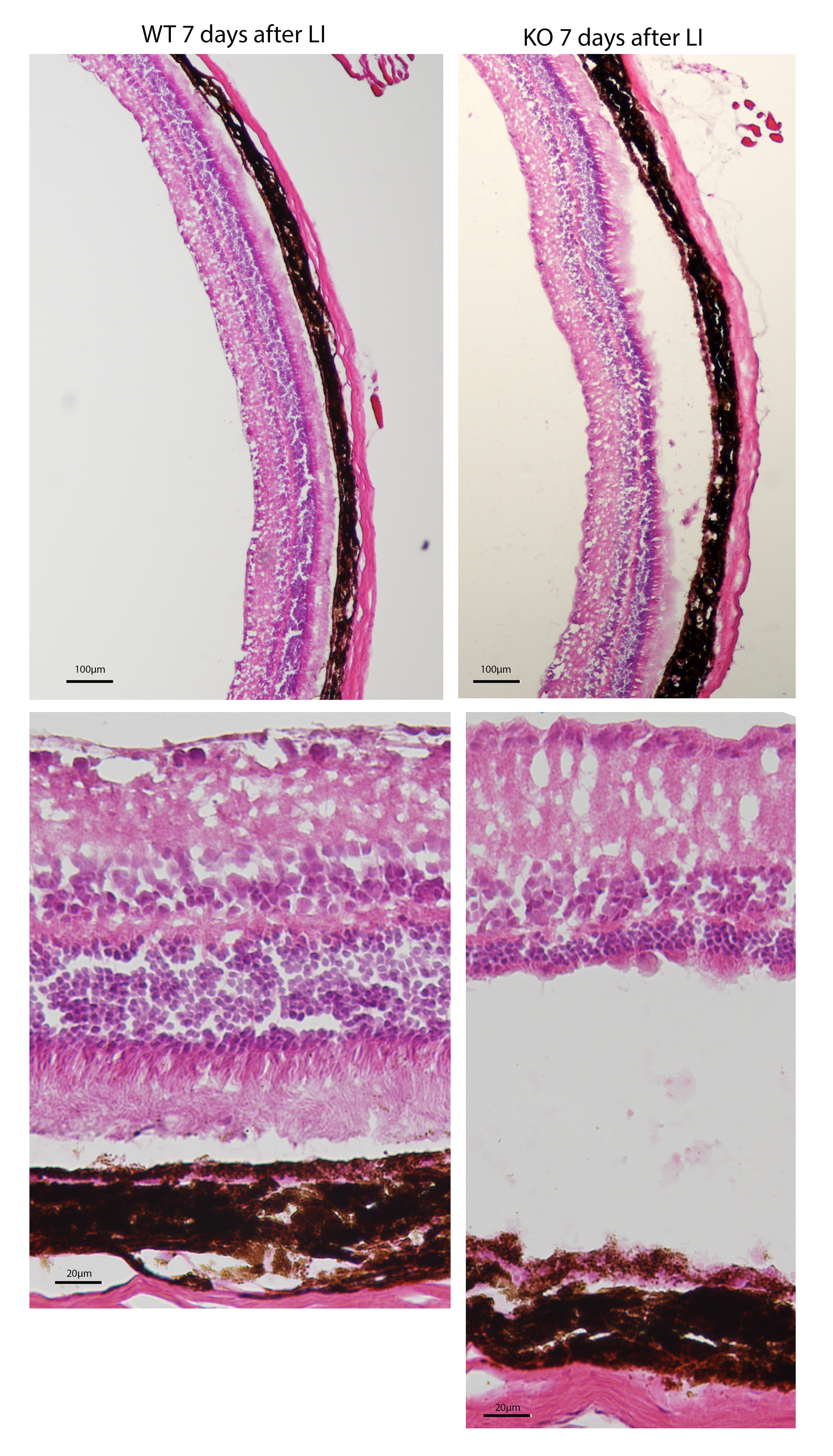

Supplement: Supplementary file 1 [file ijms-23-13108-s001.zip › figure S4.jpg]
